# The #orthorexia community on Instagram

**DOI:** 10.1007/s40519-021-01157-w

**Published:** 2021-04-09

**Authors:** Martina Valente, Sophie Renckens, Joske Bunders-Aelen, Elena V. Syurina

**Affiliations:** Athena Institute, Faculty of Science, Vrije Universiteit Amsterdam, De Boelelaan 1085, 1081 HV Amsterdam, The Netherlands

**Keywords:** Orthorexia nervosa, Instagram, Online communities, Content analysis, Social media

## Abstract

**Purpose:**

This mixed-methods study delved into the relationship between orthorexia nervosa (ON) and Instagram.

**Methods:**

Two quantitative data sources were used: content analysis of pictures using #orthorexia (*n* = 3027), and an online questionnaire investigating the experience of ON and the use of Instagram of people sharing ON-related content on Instagram (*n* = 185). Following, interviews (*n* = 9) were conducted with people posting ON-related content on Instagram and self-identifying as having (had) ON.

**Results:**

People who share ON-related content on Instagram were found to be primarily young women (questionnaire = 95.2% females, mean age 26.2 years; interviews = 100% females, mean age 28.4 years), who were found to be heavy social media users and favor Instagram over other platforms. Questionnaire respondents agreed in defining ON as an obsession with a diet considered healthy, with bio-psycho-social negative consequences, though those who self-identified as having (had) ON were more likely to point out the negative impairments of ON. Interviewees deemed Instagram partially responsible for the development of ON. Instead, they agreed that Instagram encourages problem realization. Content analysis showed that ON is encoded in pictures of ‘food’, ‘people’, ‘text’ and ‘other.’ Interviewees revealed that they started posting to recover, share information, help others, and they felt inspired to post by other accounts. A sense of belonging to the #orthorexia community emerged, where people share values and ideals, and seek validation from others.

**Conclusion:**

Conversations around #orthorexia on Instagram generate supportive communities aiding recovery. Individuals use Instagram for helping others and themselves recovering from ON. Understanding how people help each other, manage their health, cope with symptoms, and undertake recovery can inform the implementation of therapeutic interventions for ON.

**Level of evidence:**

Level III, evidence obtained from well-designed cohort or case–control analytic studies.

## Introduction

Eating disorders (EDs) are characterized by a disturbed eating behavior leading to an altered consumption of food, resulting in impairments in bio-psycho-social functioning [1]. Main EDs in the fifth edition of the Diagnostic and Statistical Manual of Mental Disorders include anorexia nervosa (AN), bulimia nervosa (BN), and binge eating. People may engage in unhealthy eating behaviors without meeting the clinical criteria for specific EDs; scholars refer to these as ‘disordered eating behaviors’ [2, 3].

A disordered eating behavior that has recently gained scientific interest is orthorexia nervosa (ON). ON is described as an obsession with healthy eating, in which health concerns lead to an extreme preoccupation with healthy food [4, 5]. The persistent and disturbing thoughts characterizing the obsession impact physiological, psychological, and social wellbeing [5]. Individuals with ON appear to be conditioned by a sociocultural context that promotes the consumption of organic whole foods, allows dedicating to food planning and preparation in terms of availability of time and money, and sees the spread of the clean eating trend [6]. In addition to sociocultural factors, ON is largely influenced by psychological factors, such as perfectionism, drive for thinness and fear of losing control [6]. Although more and more research is being conducted on ON, there are still many unknowns. For example, the prevalence rate of ON in the general population seems to fall between 1 and 7% [7], but scholars warn against relying on these estimates because they were calculated using debatable diagnostic tools [8, 9].

Overall, the knowledge on risk factors and developmental pathways of EDs and disordered eating behaviors such as ON lists some gaps, and researchers have yet to ascertain the contribution of certain factors in triggering, preventing and/or treating disordered eating practices; one of these factors being the Internet.

With the advent of the Internet, scholars have increasingly turned their attention to how information communication technologies influence disordered eating practices [2]. Some studies have assessed the potential benefits of Internet-based interventions for people with EDs [e.g. 10]; others have reviewed the risks of visiting pro-ED websites [e.g. 11]. In general, past research has focused on the impact of platforms which specifically target individuals with EDs; only recently attention has been turned to the impact of general-purpose online spaces, like social media [2].

Social media appear to have a dual role: they can aid recovery as well as stimulate pro-EDs thoughts [2, 12, 13]. Positive effects of social media include facilitating the acquisition of information about recovery, allowing tracking of one’s own recovery, and reducing stigma [2, 14, 15]. These positive effects are counterbalanced by negative effects, like triggering EDs symptoms or promoting comparison [2, 16, 17].

Additionally, communities are created on social media that influence disordered eating behaviors [18, 19]. These are groups of individuals who have a common interest, share and consume content related to that interest, and learn from and with each other [10]. Online communities are not static, since they are a product of participation rather than membership structures. For this reason, they are referred to as Communities of Practice (CoPs) [11]. Beliefs and narratives concerning disordered eating behaviors shared within CoPs may reinforce disordered eating habits, or enhance recovery.

In particular, there seems to be a link between the emergence, development and nature of ON and social media. After Instagrammer Jordan Younger confessed to suffer from ON, the concept of ON began spreading among the general population online [20]. This contributed to ON being defined a ‘cyberpathy,’ namely a condition propagated on social media by a system of social influence and cultural contagion [21].

Being the platform of choice of the healthy eating community [22], Instagram has been the social media platform most studied with regard to ON. To date, two studies have been conducted on the association between Instagram and ON: Turner and Lefevre conducted an online survey investigating demographics, social media use, dietary choices and levels of ON by using the ORTO-15 test, delivered to a sample of 680 females social media users (mean age = 24.70), concluding that higher Instagram use is linked to increased ON symptoms [22]; Santarossa et al. performed an exploratory analysis of Instagram posts using #orthorexia, and identified a relatively small but supportive community encouraging recovery [15]. These studies allow to grasp a connection between ON and Instagram. However, a broader overview of how and why people interact on Instagram regarding ON is lacking.

This study aims to contribute to understanding the conversation around ON on Instagram, by conducting a mixed-methods investigation into the relationship between ON and Instagram. This will be done by considering three aspects: *Who* shares ON-related content on Instagram? *What* type of content is shared on Instagram about ON? *Why* do individuals share ON-related content on Instagram?

The choice of using mixed-methods has been made because it seeks elaboration, illustration and clarification of different types of results,—i.e. content analysis data, which provided an unbiased source of information on the ON-related content shared on Instagram, and questionnaire and interviews findings, which allowed for an in-depth understanding of the ON-related use of Instagram, and the reasons for sharing such content.

## Methods

### Study design

This study used mixed-methods, with a sequential explanatory design. Quantitative data were obtained using (1) a content analysis of #orthorexia pictures, providing descriptive information about the type of Instagram pictures, and (2) an online questionnaire among people posting about ON (both self-identifying as having (had) ON or not, based on a provided definition). Qualitative data collection followed in the form of semi-structured interviews with people who posted about ON on Instagram and who self-identified as having (had) ON.

### Quantitative component

#### Instagram content analysis

##### Data collection

In total, 17,000 pictures using #orthorexia posted in March 2019 were downloaded using the command-line application Instagram-scraper (https://github.com/rarcega/instagram-scraper). A sub-sample of 3.027 pictures was randomly selected for analysis.^1^

##### Data analysis

After an initial look at the pictures and consultation of literature [e.g. 18, 23], a general codebook was developed. This was updated during the coding process, allowing the emergence of new codes as unexpected categories were encountered. The final codebook included 27 codes to categorize the pictures, such as salad, sweet food, individual without a face, and mirror selfie. One code was assigned to each picture, and pictures were coded based on the most prominent object they depicted. Subsequently, these pictures were placed into four main categories (‘food’, ‘people’, ‘text’ and ‘other’). Descriptive statistics were used to report the results of content analysis.

##### Ethical considerations

To minimize harm and to assure anonymity of the people posting about ON it was decided to not report any Instagram pictures or (user)names in this article. Publication of these data would allow future possible (mis)use of the analysed data that is beyond the control of the authors of the present study. Only anonymized aggregated data are reported. Although the Instagram pictures and accounts used are publically available, it is questionable to what extent these are intentionally public [24, 25]. Content analysis studies have dealt with this differently (e.g. Ging and Garvey [18] published Instagram pictures in their article on pro-ana and thinspiration content, whereas Santarossa et al. [15] did not display any pictures in their article on #orthorexia-content). In the present study, we have taken the six guiding principles for Internet research by Markham and Buchanan [26] into account to comply with ethical standards.

#### Online Questionnaire

##### Data collection

An invitation for the questionnaire was sent through a direct message on Instagram to people using ON-related hashtags, e.g. #orthorexia, #orthorexiarecovery. A post promoting the questionnaire was also shared on Instagram through a project-related account. The questionnaire was developed using Qualtrics Survey, and consisted of six parts: (i) demographics (e.g. "Which gender do you currently identify with?"), (ii) opinion and knowledge about ON (e.g. "Could you describe what orthorexia is to you, in a sentence or two?"), (iii) personal history (e.g. "Have you ever followed any (special) diets?"), (iv) contextual dynamics (e.g. "On a scale of 1–10, how much social pressure do you feel to eat healthy in general?"), (v) experiences with ON (e.g. "How did your experience with orthorexia influence your relationship with friends and family?"), and (vi) social media and Instagram use related to ON (e.g. "Have you ever posted about orthorexia on Instagram?"). For this study, parts i, ii, and vi were used. Parts iii, iv and v were used exclusively in the sister study of Valente et al. [12].

##### Data analysis

Questionnaire results were analyzed using IBM SPSS Statistics 23 and StataIC 15. Inclusion criteria were: provided consent to participate, completed the questionnaire, and aged 16 or older. Descriptive statistics were used to analyze demographics, opinion and knowledge about ON, and social media and Instagram use. Open-ended questions were analyzed in Excel using open and axial coding. Fisher’s exact test, Mann–Whitney U test and Pearson’s chi-squared test were used to calculate associations between variables and self-identification with ON vs. non-self-identification. All tests were two-tailed; the significance level was set to alpha 0.05.

### Qualitative component

#### Interviews

##### Data collection

**Fig. 1:**
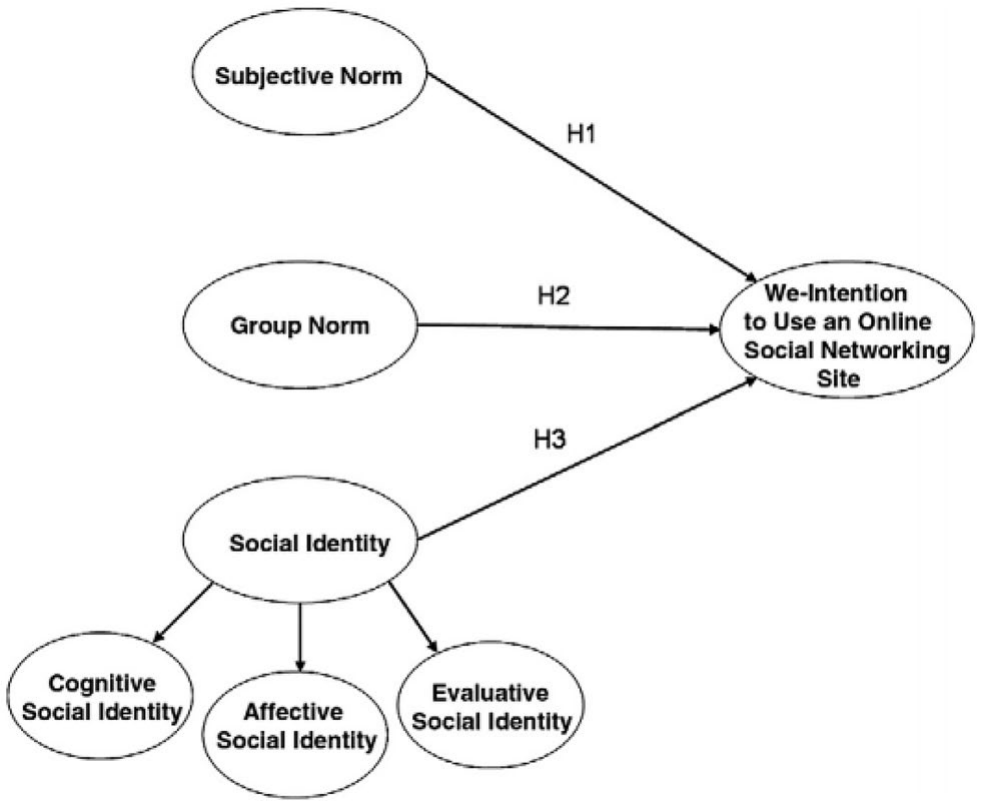
Model describing intention to use an online social network [27]

Questionnaire respondents who filled in their email address were invited to participate in an interview. Interviewees had to be at least 16 years old, have provided consent to participate, post about ON, and self-identify as having (had) ON. Interviews were conducted in English through several platforms (Zoom, Skype, FaceTime, WhatsApp). Written consent was obtained via email, and, with permission, interviews were audio-recorded. The interview, which was based on the model describing the intention to engage in online social networking from Cheung and Lee [27] (Fig. 1), aimed at gaining insight into what content the participant had shared about ON, why this content was shared, and how Instagram influenced the onset and development of ON.

##### Data analysis

Interviews were manually transcribed and coded in Atlas.ti 8 using open and axial coding. To identify and classify intentions to post ON-related content on Instagram, the analysis relied on the model describing the intention to engage in online social networking from Cheung and Lee [27] (Fig. 1). This model includes three modes of social influence: social identity, group norm, and subjective norm. Social identity refers to the self-awareness of one’s membership in a group and consists of three second-order factors: cognitive, affective, and evaluative social identity. Group norm is about the resemblance of one’s values, beliefs, and goals to those of other group members. The last mode consists of subjective norms and refers to the need for approval from significant others [27].

## Results

Results from the content analysis, questionnaire and interviews are reported in an integrated way, following a who-what-why structure: first, an overview of questionnaire and interview samples, and their characteristics, is provided (who); followed by an exploration of the type of ON-related content shared on Instagram (what); last, motivations to share ON-related content on Instagram are presented (why).

### Who shares ON-related content on Instagram?

#### Questionnaire sample

In total, 248 individuals filled out the questionnaire; 63 responses (25.4%) were excluded because users did not meet inclusion criteria, leaving 185 participants (74.6%). Among those, 124 claimed to post about ON on Instagram (67.0%); therefore, the majority of calculations were performed on this sub-sample. Most of the participants who posted about ON on Instagram were female (95.2%), with a mean age of 26.2 years. Among 30 nationalities, American (45.2%) and British (14.5%) were reported most frequently (Table 1).

**Table 1:** Demographics of questionnaire participants and sub-group of ‘posters’

Variables	All participants (*n* = 185)	Posters (*n* = 124)
Sex at birth^a^		
Male	8 (4.3%)	6 (4.8%)
Female	177 (95.7%)	118 (95.2%)
Intersex	–	–
Prefer not to answer	–	–
Current gender^a^		
Male	8 (4.3%)	6 (4.8%)
Female	177 (95.7%)	118 (95.2%)
Intersex	–	–
Other	–	–
Prefer not to answer	–	–
Age (in years); mean (SD)	25.3 (8.1)	26.2 (8.1)
Nationality^a,^^b^		
American	63 (34.1%)	56 (45.2%)
Australian	8 (4.3%)	7 (5.6%)
British	42 (22.7%)	18 (14.5%)
Canadian	11 (5.9%)	11 (8.9%)
Dutch	13 (7.0%)	2 (1.6%)
Russian	5 (2.7%)	4 (3.2%)
Other	53 (28.6%)	34 (27.4%)

^a^Number (%)

^b^Multiple answers were possible

#### Interview sample

Twenty-four individuals were invited for an interview and could choose to participate in an interview for this study and/or for a sister study on the developmental pathway of ON [12]. Of these, seven agreed to participate in an interview for this study, ten chose to interview for the other study, two were interviewed for both studies, four did not respond, and two were no longer willing to participate. The response rate for the interview was therefore 37.5% (*n* = 9).

These were all individuals self-identifying as having (had) ON who posted about ON on Instagram, all female (*n* = 9; 100%), with a mean age of 28.4 years (range 17–55). United States of America (*n* = 6; 66.7%) was the most prevalent country of residence, and the other countries were Switzerland (*n* = 1; 11.1%), United Kingdom (*n* = 1; 11.1%) and Canada (*n* = 1; 11.1%).

#### Knowledge and opinions about ON

When asked about the source for learning about ON, 33.3% of questionnaire respondents reported social media, followed by health professionals (27.4%). The majority of questionnaire participants (79.8%) knew that ON is not an official diagnosis, 87.9% believed ON should become an official diagnosis, 87.1% self-identified as having (had) ON, and 91.1% knew someone who might have ON (Table 2).

**Table 2:** Knowledge and opinion of questionnaire participants about ON

Variables	Participants (*N* = 124); *N* (%)
First source of ON knowledge^a^	
Social media	39 (33.3%)
Health professional	32 (27.4%)
Website	16 (13.7%)
Other	10 (8.5%)
Blog	7 (6.0%)
Book	4 (3.4%)
Television	4 (3.4%)
Friend/family	3 (2.6%)
Television	4 (3.4%)
News(paper)	2 (1.7%)
Know that ON is not an official diagnosis	
Yes	99 (79.8%)
No	25 (20.2%)
ON should be an official diagnosis	
Yes	109 (87.9%)
I am not sure	9 (7.3%)
No	6 (4.8%)
Self-identify as having (had) ON	
Yes	108 (87.1%)
No	16 (12.9%)
Know someone who might have ON	
Yes	113 (91.1%)
No	11 (8.9%)

^a^*N* = 117

##### Definition of ON

Questionnaire respondents sharing ON-related content on Instagram were asked to describe what ON was to them. The analysis shows that 66.9% defined ON as an *obsession*. Adjectives emphasizing the behavior were *unhealthy* (24.0%) and *extreme* (7.4%). The obsession was described as directed towards *eating healthy* (38.0%), *eating clean* (20.7%), *healthy food* (19.8%), or *purity* (9.1%), with 14.9% reporting *being healthy* as the ultimate goal. *Restricting* the range of foods was mentioned by 17.4% of respondents, and *exercise* was associated with ON by 14.9% of respondents. Among the negative consequences of ON were *anxiety* (10.7%), *interference with daily life* (9.9%), and *psychological impairment* (8.3%). Notably, 8.3% of respondents called ON an *eating disorder*, while 9.1% did not use any terms related to eating. Comparing descriptive statistics of the terms used in the definitions of ON revealed that the definitions provided by those who self-identified as having (had) ON and the definitions provided by those who did not were similar, except for the fact that terms *extreme*, *psychological impairment* and *interference with daily life* were only mentioned by those who self-identified as having (had) ON.

#### Social media use

For questionnaire respondents, Instagram was the most used social media platform (96.2%) and the highest ranked according to the time spent on it. These respondents spent a considerable amount of time on social media; e.g. approximately one in four questionnaire participants (28.6%) used social media more than 3 h a day. No significant difference was found in hours of social media use between people who did and who did not self-identify as having (had) ON (*p* = 0.766) (Table 3).

**Table 3:** Social media use of questionnaire participants

Variables	Participants (*N* = 124); *N*(%)
Age starting social media use (in years); mean (SD)	16.2 (6.0)
Social media platforms used^a^	
Instagram	124 (100.0%)
Facebook	105 (84.7%)
YouTube	81 (65.3%)
Pinterest	64 (51.6%)
Snapchat	50 (40.3%)
Twitter	33 (26.6%)
LinkedIn	33 (26.6%)
Tumblr	17 (14.5%)
Flickr	–
Ranking social media platforms used (scale 1–8); mean (SD)	
Instagram	1.2 ± 0.5
Facebook	2.6 ± 1.1
YouTube	2.8 ± 1.1
Snapchat	3.6 ± 1.4
Twitter	3.9 ± 1.5
Pinterest	4.0 ± 1.2
LinkedIn	4.8 ± 1.4
Tumblr	5.4 ± 1.5
Flickr	–
Hours of social media use per day	
< 30 min	2 (1.6%)
30–60 min	28 (22.6%)
1–3 h	59 (47.6%)
> 3 h	35 (28.2%)

^a^Multiple answers were possible

##### Influence of Instagram on the development of ON

Influence of other people’s posts on the development of ON was investigated in the interviews. Two interviewees were not on Instagram when they developed ON. The majority of the other interviewees (*n* = 5) said that Instagram affected ON development to a certain extent, however, it was not the main cause. Content that was considered most harmful concerned diets, especially clean eating: “There are some really triggering ones [accounts], because people think it is just a diet. Maybe you heard about those pro-ana groups, there is also some people that are pro-orthorexia and I think it is really triggering so I don’t look it up myself because I don’t want to get triggered.” (P2, 17 years). Instagram use also had positive effects through problem realization: “I think Instagram actually helped me because it helped me realize what I was doing wasn’t normal.” (P3, 19 years).

### What type of content is shared on Instagram about ON?

The type of ON-related content on Instagram was investigated through both a content analysis and the questionnaire. Of 3027 pictures analyzed in the content analysis, 42.5% displayed food, 18.1% people, 34.8% text, and 4.6% were categorized as ‘other.’ In the category ‘food,’ pictures of savory and sweet food were most common: respectively, 18.3% and 14.6%. Most pictures in the category ‘people’ depicted an individual whose face and other body parts were visible (7.6%). Art pictures were most highly represented in the category ‘other’ (Table 4).

**Table 4:** Content analysis of a subsample of #orthorexia pictures (*n* = 3027)

Main code	Detailed code	*N* (%)
Food (*n* = 1287; 42.5%)	Savory food	554 (18.3%)
	Sweet food	443 (14.6%)
	Pizza/pasta	72 (2.4%)
	Food and drink	58 (1.9%)
	Fruit and vegetable	58 (1.9%)
	Salad	54 (1.8%)
	Drink	48 (1.6%)
Text (*n* = 1054; 34.8%)	Text	1054 (34.8%)
People (*n* = 548; 18.1%)	Individual including face	231 (7.6%)
	Group	74 (2.4%)
	Face	71 (2.3%)
	Mirror selfie	71 (2.3%)
	Multiple photos individual	41 (1.4%)
	Individual without face	23 (0.8%)
	Body parts	22 (0.7%)
	Exercise	15 (0.5%)
Other (*n* = 138; 4.6%)	Art	63 (2.1%)
	Object	32 (1.1%)
	Scenery	18 (0.6%)
	Room	6 (0.2%)
	Dishware	5 (0.2%)
	Multiple photos other	5 (0.2%)
	Dog	3 (0.1%)
	Map	2 (0.1%)
	Cat	2 (0.1%)
	Art exhibition	1 (< 0.1%)
	Horse	1 (< 0.1%)

Questionnaire respondents also answered what content they share on Instagram: people (50.0%), food/recipes (39.5%), text (35.5%) and other (39.5%). Chi-squared test was used to assess differences between questionnaire respondents who self-identified as having (had) ON and those who did not in regard to their ON-related Instagram use. People who self-identified as having (had) ON were significantly more likely to post about ON (*p* < 0.001), to search for #orthorexia (*p* = 0.006), and to follow ON-related accounts (*p* = 0.008). They were also significantly more likely to post content belonging to the categories ‘food’, ‘people’ and ‘text,’ as compared to people who did not self-identify (all *p* < 0.05); the latter group was significantly more likely to post content in the ‘other’ category (*p* < 0.0001) (Table 5).

**Table 5:** Crosstabulation of Instagram use and self-identification

Variable		Self-identify as having (had) ON; *N* (%)			*p* value
		Yes	No	Total	
Posted about ON on Instagram	Yes	108 (76.2%)	16 (45.7%)	124 (70.1%)	< 0.0001
Posted with #orthorexia on Instagram^b^	Yes	103 (95.4%)	16 (100%)	119 (96%)	1.000
Content posted about ON on Instagram^b,^^c^	Food	49 (45.4%)^a^	0 (0%)^a^	49 (39.5%)^a^	0.001
	People	59 (54.6%)^a^	3 (18.8%)^a^	62 (50.0%)^a^	0.007
	Text	42 (38.9%)^a^	2 (12.5%)^a^	44 (35.5%)^a^	0.04
	Other	35 (32.4%)^a^	14 (87.5%)^a^	49 (39.5%)^a^	< 0.0001
Look for #orthorexia content on Instagram	Yes	73 (51.4%)	9 (25.7%)	82 (46.3%)	0.006
Follow #orthorexia on Instagram^d^	Yes	11 (15.1%)	1 (11.1%)	12 (14.6%)	1.000
Follow ON-related Instagram accounts	Yes	63 (44.4%)	7 (20.0%)	70 (39.5%)	0.008

^a^% of cases

^b^Only answered by participants who posted about ON on Instagram

^c^Multiple answers were possible

^d^Only answered by participants who looked for #orthorexia on Instagram

### Why do people share ON-related content on Instagram?

#### Reasons to start posting

The interviewees mentioned a drive to recover (*n* = 4) most frequently as a reason to start posting because posting stimulated a sort of ‘peer-pressure’ to continue recovering: “I really needed to talk about this to make myself accountable for my recovery. Now people know I am doing this, so I have to keep doing it.” (P8, 55 years). Another reason that was brought up by some of the interviewees was the urge to share knowledge about ON (*n* = 2). One interviewee mentioned to have started posting about ON because other people were doing so: “In my Instagram explore page I started seeing posts about it [ON] and I wanted to create my own.” (P9, 17 years).

#### Intentions to post content about ON on Instagram

Intentions for posting ON-related content that were investigated in the interviews have been classified according to Cheung and Lee’s model of intention to engage in online social networking [27] (Fig. 1).

##### Social identity

The feeling of belonging to a certain ‘orthorexia community’ is found to be an important driver for posting about ON on Instagram. According to all interviewees (*n* = 9) there is a community around ON: “I: ‘Do you think there is some sort of community on Instagram talking about these kinds of things?’ R: ‘Yes, absolutely. Absolutely. I found some really amazing people through Instagram. It is fantastic.’ (P8, 55 years). Communities that were mentioned revolved around body positivity, intuitive eating, mental health and wellness. The three dimensions of social identity, i.e. cognitive, affective and evaluative social identity [27], were all found to contribute to the intention to post about ON on Instagram.

Cognitive social identity concerns the perception that community members are distinct from people outside the community, who do not understand what ON encompasses. This was recognized by six of the interviewees: “As a society, this is not something we talk about or are aware of, so when I am able to read [ON-related content] I realize I am not the only one that feels that way.” (P3, 19 years).

Affective social identity refers to the emotional involvement in the community and was mentioned by the majority of interviewees (*n* = 6) in the form of positive feelings deriving from belonging to the community: “It makes me feel good. I enjoy it.” (P4, 41 years).

With regard to evaluative social identity, membership of a community around ON is validating and reinforces self-worth according to six of the interviewees: “Not only I feel I am being validated for the work that I do, but also if I am having a bad body day, or I am having doubts about recovery, I can talk about that with these people. That really boosts me up a lot, that is great.” (P8, 55 years).

##### Group norm

All interviewees (*n* = 9) perceived that there are shared values within the community: “I just really felt I could relate to what they shared and I thought I could contribute to that.” (P9, 17 years). Interestingly, it emerged that, although people felt less alone because others experienced the same ‘journey’, they also noted how behaviors and experiences might differ from one person to the other.

##### Subjective norm

The subjective norm is the need for approval from other members, mostly expressed in the form of likes and comments on Instagram. The majority of interviewees (*n* = 6) felt that other people’s appreciation is important: “If I see significant less likes I start to wonder why. Do people not like me as much, or why do people like this person more? I start thinking those negative thoughts.” (P5, 28 years). On the contrary, a few participants (*n* = 3) mentioned not to care about approval from others: “I don’t need a certain number to feel good about the post. If it doesn’t hit a certain number, I am not going to delete it.” (P9, 17 years).

#### Aim of the posts about orthorexia nervosa

Interviewees also mentioned the drive to reach a certain goal with posting about ON on Instagram. Two major goals were found: to raise awareness on how the pursuit of health can become harmful (*n* = 8), and to support other people who are struggling (*n* = 8): “I wanted to show other people that even if it is hard, you can get out of it. It needs some hard work, you need to face your fears to get out of it, but it is worth it and you can do it.” (P2, 17 years). Another goal mentioned by five interviewees was to help themselves with recovery: “It keeps me accountable and reminds me of why I want to stay healthy and not go back down on an eating disorder path.” (P9, 17 years).

## Discussion

By using mixed methods, this study collected information on who shares ON-related content on Instagram, what type of content is shared, and why such content is shared. The findings of this study ultimately enrich the literature on social media and mental health.

In the attempt to answer the question: “Who shares ON-related content on Instagram?”, this study found that people who share ON-related content on Instagram are primarily young women (questionnaire = 95.2% females, mean age 26.2 years; interviews = 100% females, mean age 28.4 years), who were found to be heavy social media users and favor Instagram over other platforms. One in three came to know about ON on social media. Their definition of ON is in line with the literature [5], though those who self-identify as having (had) ON are more likely to use words like ‘psychological impairment’ or ‘interference with life’. Those who self-identify as having (had) ON believe that Instagram might have an impact on ON, but it is not the primary cause. Instead, Instagram is considered to promote problem realization. Following, this study answered the question: “What type of content is shared on Instagram about ON?”, by finding that ON is mostly encoded in pictures of food, followed by pictures of text and people, and least often in ‘other’ pictures. Finally, this study sought to answer the question: “Why do people share ON-related content on Instagram?”, and it was found that individuals sharing ON-related content on Instagram post to recover, share information and help others. Individuals feel inspired to post by other accounts, indicating a potential ‘peer emulation’ process. An overall sense of belonging to the #orthorexia community emerged, where individuals share values and ideals and seek validation from others.

By sharing stories, values, and advices individuals sharing ON-related content on Instagram aggregate around #orthorexia, thus creating CoPs. These are ‘safe spaces’ where to share sensitive content and personal stories. Within these CoPs, individuals tell their stories and contribute to the social construction of ON [28]. Furthermore, individuals share common beliefs and ideals, which seem to reveal an ideological ‘rebellion’ against diet culture and clean eating more broadly. These spontaneous aggregations resemble a primate stage of peer support groups since individuals feel empowered in talking about their health and providing help to others [29].

Individuals seem to emulate each other through interaction on Instagram; e.g. one individual shares ON-related content because she was inspired by another user. This hints at a sort of social contagion [30], where community members would imitate each other’s online behavior. While in this case the contagion is positive, most of the studies investigating social contagion reveal a negative contagion. For example, evidence suggests that social contagion contributes to the spread of EDs among young women, with social media intensifying this process [23]. Given that social media have become increasingly prominent in young people’s lives, we encourage future research to further assess the potential positive and negative effects that these platforms might have on EDs.

Our findings are in line with those of Santarossa et al. [15], who found a small supportive community, mostly composed of females, interacting on Instagram about ON. Yet, our results go one step further, by exploring intentions to share such supportive content, painting a more comprehensive picture. Our results also corroborate those of Turner and Lefevre [22]. Although we found a predominantly positive conversation around ON, respondents agreed that diet- and clean eating-related content could trigger ON. Similarly, Andalibi et al. in their investigation into #depression on Instagram shed light on the presence of great social support, a strong sense of community and little sharing of pro-disease behaviors [31]. Finally, comparing the results of the present study with the findings of a recent investigation into the #orthorexia conversation on Twitter leads to identifying interesting differences. First of all, on Twitter the most prominent actors belong to the professional sphere (e.g. dietitians, researchers), with a very small number of people suffering from the disorder actually joining the conversation. The ON-related conversation on Twitter has been proven to bring about the medicalization process of ON, and the network analysis has shown that actors engaging in the ON-related conversation on Twitter appear isolated from each other, with no formation of peer-support communities [32].

Future research should be conducted on the role of CoPs on recovery from EDs. Although we found a positive ‘orthorexia community’, our findings shed light on a search for validation from other community members. It may be useful to understand if these communities could be dangerous as they might stimulate peer pressure. A second recommendation for future research concerns the topic of identity. What we noticed is that individuals seemed to ‘melt’ their identities to that of the community: a shift from a ‘orthorexia identity’ to a ‘communitarian identity’. Going deeper into the role of identity during recovery from ON can inform treatment strategies.

While this study has strengths (e.g. the use of three methodologies; an in-depth qualitative investigation), it also has limitations (e.g. small sample size of the qualitative sample; lack of information on current or past ED psychopathology; impossibility for the present findings to be generalized to the general population, because of greater proportions of American and British participants, disproportion between genders, predominance of young individuals and lack of assessment of the severity of their ON-related psychopathology).

In conclusion, this study found that conversations around #orthorexia on Instagram generate supportive communities aiding recovery. Individuals use Instagram as a tool for helping others and themselves recovering from ON. Understanding how people help each other, manage their health, cope with ON symptoms and undertake recovery can inform therapeutic interventions.

*What is already known on this subject?*

Previous studies suggested an association between Instagram and ON, but only one study explored such association [15].

*What your study adds?*

This study showed who shares ON-related content on Instagram. It explored how is ON encoded on Instagram. Last, it provided information on the intentions for sharing ON-related content on Instagram.

## Data Availability

The data that support the findings of this study are available from the corresponding author upon reasonable request.

## Footnotes

1 Authors chose to sample 17% of the total number of pictures, as this number of pictures, i.e. 3.027, seemed manageable for manual coding.
